# Two Cases of Distant Metastasis After Mastectomy for Breast Ductal Carcinoma In Situ

**DOI:** 10.7759/cureus.59655

**Published:** 2024-05-04

**Authors:** Takeru Hamaoka, Hiroko Bando, Mai Okazaki, Akiko Iguchi-Manaka, Hisato Hara

**Affiliations:** 1 Department of Breast and Endocrine Surgery, University of Tsukuba Hospital, Ibaraki, JPN; 2 Institute of Medicine, Breast and Endocrine Surgery, University of Tsukuba, Ibaraki, JPN

**Keywords:** recurrence, breast cancer, mastectomy, distant metastasis, ductal carcinoma in situ

## Abstract

While the prognosis for ductal carcinoma in situ (DCIS) of the breast is generally excellent, distant metastasis after appropriate local treatment is extremely rare. We experienced two cases of distant metastasis after mastectomy for breast ductal carcinoma in situ. In both cases, the surgical margins were negative, the sentinel nodes were negative for metastasis. The first case was a 67-year-old woman who developed lung metastases four years after mastectomy for high-grade DCIS. The second case was a 34-year-old woman with intermediate-grade DCIS who developed intraductal recurrence localized to the nipple two years after the initial nipple-sparing mastectomy and multiple lung and liver metastases six months later. Both cases developed distant metastases despite appropriate local treatment, without preceding or concurrent invasive local recurrence. Although the probability of distant recurrence is low, it is important to inform patients about the risk of recurrence.

## Introduction

In general, the prognosis of ductal carcinoma in situ (DCIS) is excellent. The risk of local recurrence is between 1.3% and 4.9%, although it depends on the adjuvant treatment, such as radiation treatment and/or endocrine treatment [1]. According to the Van Nuys Prognostic Index and recent studies, the risk factors for local recurrence are young age, a large tumor, positive surgical margins, high nuclear grade, comedo necrosis, and positive human epidermal growth factor 2 (HER2) status [2-5].

Distant metastasis from DCIS after appropriate local treatment (surgery) is extremely rare. In the European Organization for Research and Treatment of Cancer Trial (EORTC) 10853 trial, 1010 women were randomly assigned to receive surgery alone or surgery followed by radiotherapy [6]. In the surgery-plus-radiotherapy group, two patients developed distant metastasis after local recurrence of DCIS, and five patients developed distant metastasis without a prior local recurrence or contralateral breast cancer during a 10-year follow-up. Roses et al. have reported that among 2449 patients, nine patients developed distant metastasis without local recurrence [7]. The rarity of this condition makes it difficult to identify risk factors through research definitively, and the underlying mechanism remains unclear. Here, we report two cases of distant metastasis without preceding or simultaneous local recurrence after mastectomy for DCIS of the breast.

## Case presentation

Case one

**Figure 1:**
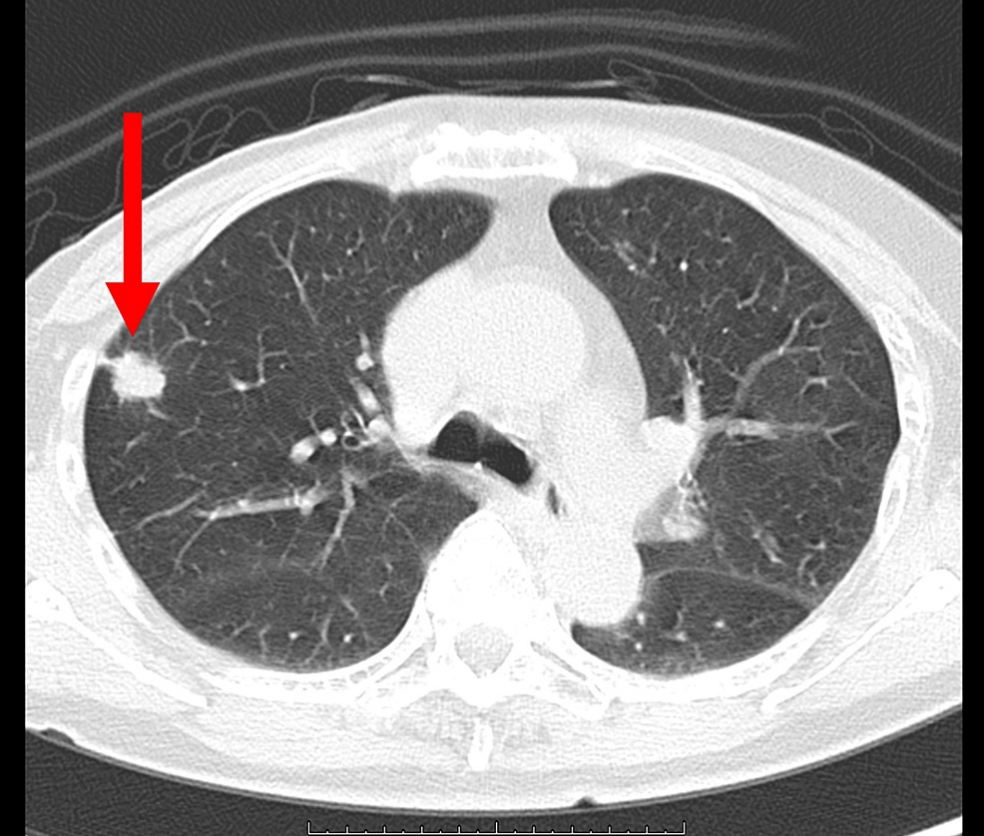
CT scan of the lung metastasis in case one A 16-mm long fine lobulated mass with spiculation can be observed in the S2b/3a region of the right upper lobe.

The first patient was a 67-year-old woman who underwent right total mastectomy. She visited the hospital because she noticed nipple discharge from the right breast. The mammograms showed heterogeneously dense breasts and fine pleomorphic segmental calcifications in the right upper outer quadrant (BiRADs Category 5). Ultrasound showed a non-mass image of a geographic hypoechoic area with hyperechoic spots in her right breast. MRI with contrast enhancement revealed a high-absorption area along the ducts. The lesion was diagnosed as high-grade DCIS through core needle biopsy, with negative status both for estrogen receptor (ER) and for progesterone receptor (PgR). She underwent right total mastectomy and sentinel lymph node biopsy, and the final pathological diagnosis of high-grade DCIS (size: 7.3 × 6.7 × 2.5 cm) with negative margins was made. ER and PgR were negative, and there was no metastasis in the resected two sentinel lymph nodes. Regular annual physical check-up was provided. Four years after surgery, a right lung tumor was observed during a routine X-ray examination for lung cancer screening in the public health system. The maximum size of the lesion was 16 mm in the S2b/3a area and it was suspected to be a primary lung cancer by CT scan (Figure 1).

**Figure 2:**
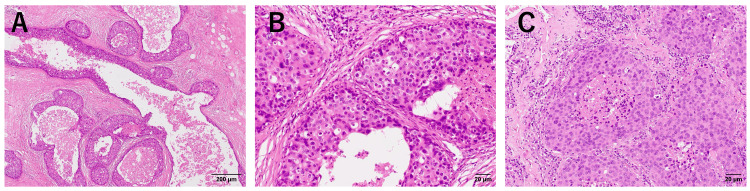
Pathology of the surgical specimens of case one A: Surgical specimen of the breast (x4); B: Surgical specimen of the breast (x20); C: Surgical specimen of the lung metastasis (x20). Tumor cells similar to the primary tumor can be observed in the lung specimen.

Partial lung resection was performed, and the pathological diagnosis revealed the tumor to be a metastatic carcinoma of breast cancer (Figure 2).

The subtype of the metastatic lung tumor was ER- and PgR- negative, HER2-positive (3+), TTF1 negative, NapsinA negative, and GCDFP-15 positive. She received a combination treatment with six cycles of trastuzumab, pertuzumab, and docetaxel, followed by trastuzumab and pertuzumab for two years. The patient is currently being followed up with no systemic treatment and has been free of cancer for nine years after recurrence. 

Case two

**Figure 3:**
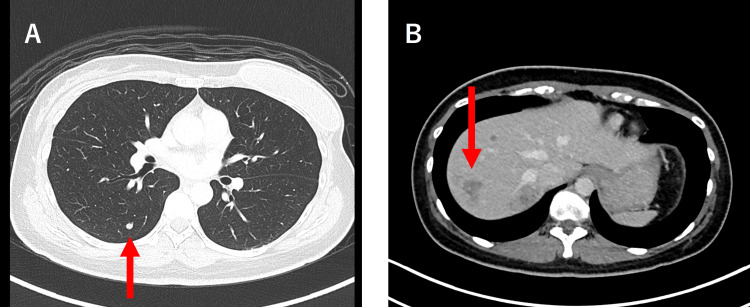
CT scan of the liver and lung metastasis of case two A: CT scan of the liver metastasis. A small nodule can be seen in the right lung (arrow); B: CT scan of the liver metastasis. Multiple low-absorption lesions of suspected multiple liver metastases of 10 mm to 30 mm in size can be observed.

The second patient was a 34-year-old woman. A breast lesion was detected during screening, and it was diagnosed as DCIS of an intermediate grade, with positive status for ER and PgR. The maximum diameter of the lesion was 1.5 cm, and the lesion was palpable at the 12 o’clock position in her left breast. The mammograms showed heterogeneously dense breasts and coarse, heterogeneous, grouped calcifications (BiRADs Category 4). Ultrasound showed an indistinct hypoechoic area with small hyperechoic spots inside and with abundant blood flow. It also showed multiple small nodules along the duct. MRI with contrast enhancement revealed a high-absorption area in her left breast. She underwent a left nipple-sparing mastectomy and immediate breast construction. The final pathological diagnosis was DCIS of an intermediate grade (size: 4.2 × 2.2 × 2.0 cm), with negative margins, ER- and PgR-positive, HER2-negative, and without metastasis in the sentinel lymph nodes. Regular physical examination at intervals of three to four months was provided without systemic treatment and two years after the surgery, bloody nipple discharge was detected from the remaining nipple. Local recurrence was suspected, and resection of the nipple-areolar complex was performed. The pathological diagnosis was DCIS recurrence in the nipple (size: 18 × 3 mm), ER- and PgR-positive, HER2-negative, with negative surgical margins. At that time, there was no evidence of regional lymph node metastasis and no systemic recurrence was observed on imaging. She started treatment with tamoxifen, but after six months, multiple hepatic tumors, lung nodules and an enlarged level III lymph node in the left axilla were found by CT scan during a regular health checkup at her workplace (Figure 3).

**Figure 4:**
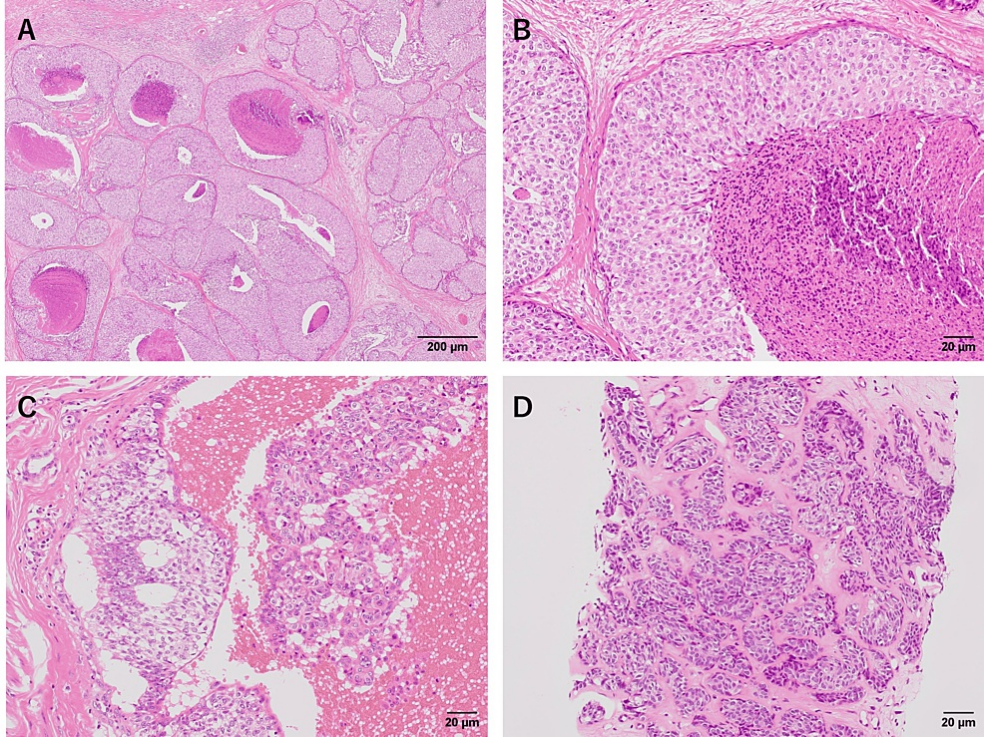
Pathology of case two A: Surgical specimen of the breast obtained from the first operation (x4); Extensive areas of tumor cells with comedo necrosis can be observed in the dilated ducts, but no invasive areas can be observed in the primary breast specimen; B: Surgical specimen of the breast obtained from the first operation (x20); C: Surgical specimen of the breast obtained from the second operation of resection of the nipple-areolar complex (x20); D: Core needle biopsy specimen of the liver metastasis (x20).

The liver and lung tumors were biopsied, and distant metastasis was confirmed (Figure 4).

The pathological features of the metastatic tumors were similar to the primary tumor, and the immunostaining showed these tumors to be ER- and PgR-positive, HER2-negative, TTF1 negative, and NapsinA negative. She started treatment with fulvestrant, a luteinizing hormone-releasing hormone agonist, and abemaciclib, which achieved a partial response. After 13 months, her liver tumor progressed, and treatment was switched to chemotherapy.

## Discussion

We have presented two cases of DCIS treated with mastectomy, which subsequently developed distant metastasis. DCIS is defined as a non-invasive precursor lesion that theoretically does not metastasize or cause death without progression to an invasive breast lesion [8]. In the American Joint Committee on Cancer Staging Manual, DCIS with microinvasion (≤ 0.1 cm in size) is also defined as DCIS. In pure DCIS, the cancer spread is limited to the breast duct without invasion through the basal membrane, so the potential of DCIS for distant metastasis without preceding invasive local recurrence is barely expected.

The underlying mechanism for the development of distant metastasis in DCIS is not completely clear. DCIS cells resemble invasive cancer cells, and despite careful histologic analysis, small areas of invasive cancer cells or foci can be missed in DCIS lesions and end up being misclassified as DCIS. In a study of DCIS cases from the Surveillance, Epidemiology, and End Results (SEER) program, the 10-year breast cancer mortality rate was 3.4% for those diagnosed between 1978 and 1983, 1.9% for those diagnosed between 1984 and 1989, and 1.1% for those diagnosed between 1988 and 2011 [9]. The decline may be due to an improved ability to distinguish between DCIS and invasive cancer. Also, since not all microinvasive lesions develop metastasis, such misclassification may not be the only reason for distant metastasis of DCIS. 

Local recurrence, especially one that now contains an invasive carcinoma component, is a known risk factor is a known risk factor for distant metastasis; however, some cases of distant metastasis without invasive local recurrence have been reported (Table 1).

**Table 1:** The rate of distant metastasis and death from DCIS * Not reported; † Not applicable; ‡ Cases initially treated at M.D. Anderson Cancer Center; DCIS: Ductal carcinoma in situ.

Author, Year of publication, reference number	Number of cases	Lumpectomy (±radiotherapy) n (%)	Mastectomy n (%)	Local recurrence n (%)	Distant metastasis after local recurrence n (%)	Distant metastasis without local recurrence n (%)	Distant metastasis without local recurrence after total mastectomy n (%)	Total distant metastasis n (%)	Deaths from breast cancer n (%)
Bijker et al., 2001 [6]	1010	1010 (100.0)	ｰ^†^	136 (13.47)	17 (1.68)	3 (0.29)	ｰ^†^	24 (2.38)	11 (1.09)
Roses et al., 2011 [7]	2449	ｰ*	ｰ*	113 (4.61)	16 (0.65)	9 (0.36)	4 (0.16)	25 (1.02)	14 (0.57)
Roses et al., 2011 [7]	2123^‡^	ｰ*	ｰ*	56 (2.63)	2 (0.09)	1 (0.04)	ｰ*	3 (0.14)	ｰ*
Rakovitch et al., 2012 [4]	213	213 (100.0)	0 (0.00)	50 (23.42)	3 (1.41)	0 (0.00)	ｰ^†^	3 (1.41)	ｰ*
Narod et al., 2015 [9]	108196	63319 (58.5)	25527 (23.6)	3312 (3.06)	395 (0.37)	517 (0.48)	112 (0.10)	ｰ*	956 (0.88)
Bernard et al., 1998 [10]	814	814 (100)	0 (0.00)	157 (19.29)	6 (0.74)	6 (0.74)	ｰ^†^	12 (1.47)	13 (1.60)

In a study of DCIS cases from the SEER program, the risk of death from DCIS was 1.3% for those undergoing mastectomy and 0.8% for those undergoing lumpectomy and radiotherapy. In this analysis, mastectomy reduced the risk of local recurrence by 75% but did not reduce the risk of dying. In another literature review, it was reported that the prevention of in-breast invasive recurrences may not prevent death from breast cancer [11]. It was also reported that among 108,196 patients with DCIS, 517 patients died of breast cancer without experiencing an in-breast invasive cancer prior to death. It can be hypothesized that breast cancer dissemination may occur during the pre-invasive stages. In 13% to 25% of DCIS cases, circulating tumor cells in the peripheral blood or the bone marrow have been reported and their frequency is comparable to that of patients with invasive disease [12-14]. These reports support the possibility of DCIS cells entering the circulating system before passing into the basement membrane. On the other hand, there are studies that have investigated the outcomes of foregoing immediate surgery in cases of low-risk DCIS, but the definition of “low risk” varies in each study [15-19]. Further research is required to facilitate a detailed risk classification of DCIS according to histology and molecular subclassifications.

According to the guidelines, radiation for DCIS patients treated with breast-conserving surgery and endocrine therapy for ER-positive DCIS is recommended or considered to reduce the risk of ipsilateral local recurrence; however, additional systemic treatment to prevent distant metastasis is not recommended for patients with DCIS after mastectomy. The National Surgical Adjuvant Breast and Bowel Project (NSABB P)-43 trial investigated the efficacy of adding trastuzumab following surgery and radiation therapy for HER2-positive DCIS patients and concluded that this was not effective [10,20].

The typical follow-up of patients with DCIS includes interval history and physical examination every six to 12 months for five years and then annually, as well as yearly diagnostic mammography of the remaining breast. Distant metastasis may not be detected by the recommended DCIS follow-up, which suggests that other modalities such as lung cancer screening or regular medical check-ups (which proved helpful in our cases) should be leveraged.

## Conclusions

In conclusion, we have presented two cases of distant metastasis in patients with DCIS following total mastectomy. DCIS is defined as a non-invasive precursor lesion, and the incidence of distant metastasis of DCIS after mastectomy is extremely low, so the risk is barely expected.

Invasive local recurrence is a known risk factor for distant metastasis after DCIS surgery, but neither occurred in our two cases. Distant metastasis of DCIS without invasive local recurrence is not clear yet. Despite careful histologic analysis, small areas of invasive cancer cells or foci could have been missed in DCIS lesions, but on the other hand, the possibility of DCIS cells entering the circulating system before passing into the basement membrane is also reported. Additional systemic treatment to prevent distant metastasis is not recommended for patients with DCIS after mastectomy, and distant metastasis may not be detected by regular check-ups for breast cancer follow-up.

In summary, our cases serve as a reminder that we should inform the patients with DCIS who underwent mastectomy about the limited but substantial risk of recurrence, and modalities such as lung cancer screening or regular medical check-ups should be leveraged. Since DCIS is a heterogeneous disease, further research is warranted to assess the risk of metastasis.
